# Crystal Structure of SARS-CoV-2 Main Protease in Complex with the Non-Covalent Inhibitor ML188

**DOI:** 10.3390/v13020174

**Published:** 2021-01-25

**Authors:** Gordon J. Lockbaum, Archie C. Reyes, Jeong Min Lee, Ronak Tilvawala, Ellen A. Nalivaika, Akbar Ali, Nese Kurt Yilmaz, Paul R. Thompson, Celia A. Schiffer

**Affiliations:** Department of Biochemistry and Molecular Pharmacology, University of Massachusetts Medical School, Worcester, MA 01605, USA; gordon.lockbaum@umassmed.edu (G.J.L.); archie.reyes@umassmed.edu (A.C.R.); JeongMin.Lee@umassmed.edu (J.M.L.); ronak.tilvawala@ku.edu (R.T.); ellen.Nalivaika@umassmed.edu (E.A.N.); Akbar.Ali@umassmed.edu (A.A.); Nese.KurtYilmaz@umassmed.edu (N.K.Y.); Paul.Thompson@umassmed.edu (P.R.T.)

**Keywords:** SARS-CoV-2, Covid-19, main protease, M^pro^, ML188, protease inhibitor, crystal structure, structure-based drug design, direct-acting antivirals

## Abstract

Viral proteases are critical enzymes for the maturation of many human pathogenic viruses and thus are key targets for direct acting antivirals (DAAs). The current viral pandemic caused by SARS-CoV-2 is in dire need of DAAs. The Main protease (M^pro^) is the focus of extensive structure-based drug design efforts which are mostly covalent inhibitors targeting the catalytic cysteine. ML188 is a non-covalent inhibitor designed to target SARS-CoV-1 M^pro^, and provides an initial scaffold for the creation of effective pan-coronavirus inhibitors. In the current study, we found that ML188 inhibits SARS-CoV-2 M^pro^ at 2.5 µM, which is more potent than against SAR-CoV-1 M^pro^. We determined the crystal structure of ML188 in complex with SARS-CoV-2 M^pro^ to 2.39 Å resolution. Sharing 96% sequence identity, structural comparison of the two complexes only shows subtle differences. Non-covalent protease inhibitors complement the design of covalent inhibitors against SARS-CoV-2 main protease and are critical initial steps in the design of DAAs to treat CoVID 19.

## 1. Introduction

Coronavirus disease 2019 (COVID-19) is caused by severe acute respiratory syndrome coronavirus 2 (SARS-CoV-2) [1]. Zoonotic transmissions of coronaviruses to humans have caused at least three major outbreaks during the past two decades; SARS in 2002 [2,3], MERS in 2012 [4], and SARS-2 in 2019–2020 [5,6]. The previous outbreaks were contained with rigorous public health interventions, and basic research and vaccine/drug developments had largely been suspended. As is now evident from the current global pandemic [7], coronaviruses pose a major threat to human health, and treatment options need to be developed not only for the current pandemic but also for future coronavirus outbreaks. In addition to the major efforts for developing vaccines around the world, direct acting antivirals (DAAs) are critical to treat vulnerable patients to decrease morbidity and mortality.

Coronaviruses are non-segmented positive-sense single-stranded RNA viruses [8]. The viral genome encodes multiple open reading frames (ORF), including ORF1a and ORF1b, which are translated by the host machinery to generate two polyproteins (pp): pp1a and pp1ab [9]. These polyproteins are cleaved by two viral proteases, Main protease (M^pro^) and the Papain-Like protease (PL^pro^), liberating 16 individual proteins (nsp1–16). These proteolysis events are essential for the SARS-CoV-2 viral life cycle [10]. Thus, these two proteases represent excellent targets for the development of DAAs. Consistent with this notion, viral proteases are well-established targets for DAAs, with FDA-approved inhibitors as part of the mainstay of treatment for both HIV-1 and Hepatitis C virus infections. Given that the entire genomes of SARS-CoV-1 and SARS-CoV-2 are 79% identical [11], as well as the fact that SARS-CoV-2 M^pro^ (SARS2-M^pro^) and SARS-CoV-2 PL^pro^ are 96% and 83% identical to their SARS-CoV-1 orthologs, respectively (Figure S1) [12], indicates that DAAs targeting either of these two proteases will likely show efficacy for a range of coronaviruses.

The current study focuses on SARS2-M^pro^, a chymotrypsin-like cysteine protease that resides in the viral non-structural protein 5 (nsp5) [13]. M^pro^ cleaves 11 sites in pp1a and pp1ab, including autoproteolysis. The 4% difference in sequence with SARS1 corresponds to 12 changes in the 306-residue enzyme. Whereas most changes occur distal to the active site, we previously established that distal mutations can significantly affect inhibitor potency [14,15,16,17,18]. Potent, selective inhibition of SARS2-M^pro^ could be a successful therapeutic for Covid-19 and future coronaviruses. Structure-based drug design is already playing a pivotal role in targeting this viral enzyme. Fragment screens have placed hundreds of small fragments around SARS2-M^pro^, in the active site and allosteric sites [19]. Continuing this quest, several publications include crystal structures of covalent inhibitors that target the catalytic cysteine [20,21,22], including one that has entered into clinical trials [23].

**Figure 1:**
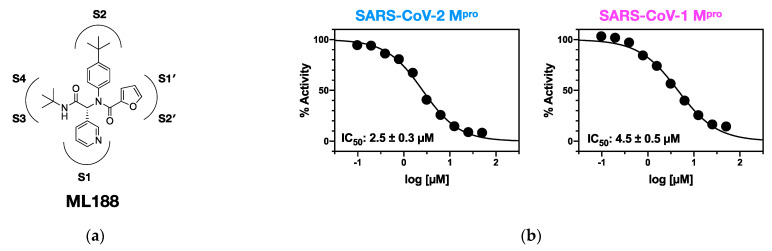
(**a**) Chemical structure of ML188. (**b**) Dose-response curves and IC_50_ values of compound ML188 against SARS2-M^Pro^ and SARS1-M^Pro^.

While covalent inhibitors have demonstrated high potency and efficacy, there is a lack of potent non-covalent inhibitors of coronavirus proteases. One potential scaffold is ML188 (Figure 1a), a non-covalent inhibitor designed to inhibit SARS1-M^pro^ [24]. ML188 was reported to bind SARS1-M^pro^ with an IC_50_ of 1.5 ± 0.3 µM and an EC_50_ of 12.9 ± 0.7 µM in cellular assays [24]. Pure ML188 has not been tested against SARS2-M^pro^, but racemic ML188 inhibits with an IC_50_ of 3.14 µM [25], which is comparable to racemic ML188 against SARS1-M^pro^ (4.8 ± 0.8 µM) [24]. Showing this scaffold’s versatility, ML188 was also found to inhibit M^pro^ from porcine epidemic diarrhea virus (PEDV) with only 17-fold less potency (IC_50_ of 25.4 ± 1.4 μM), despite SARS1-M^pro^ and PEDV-M^pro^ sharing only 45.4% sequence identity [26]. This indicates that ML188 may provide a scaffold for a robust non-covalent pan-coronavirus inhibitor.

In this study, we characterize the complex of SARS-CoV-2 M^pro^ with the noncovalent inhibitor ML188. We determine that ML188 has enhanced binding potency to SARS-CoV-2 compared with SARS-CoV-1 and solved the cocrystal structure of ML188 in complex with SARS2-M^pro^ to 2.39 Å resolution. Overall, the complexes of M^pro^ with ML188 are very similar in both proteases, but subtle differences likely contribute to the higher potency of ML188 against SARS2-M^pro^.

## 2. Materials and Methods

### 2.1. Protein Expression and Purification of SARS2-M^pro^

The SARS2-M^pro^ plasmid, kindly provided by Rolf Hilgenfeld [21], was transformed into *Escherichia coli* strain HI-Control™ BL21(DE3) (Lucigen, Middleton, WI, USA). The transformed cells were pre-cultured at 37 °C in LB medium with ampicillin (100 μg/mL) overnight, and the cell culture was inoculated into TB medium containing 50 mM sodium phosphate (pH 7.0) and ampicillin (100 μg/mL). When OD_600_ value reached ~2.0, 0.5 mM IPTG was added to induce SARS2-M^pro^ expression and the cell culture was further incubated overnight at 20 °C. Cells were harvested by centrifugation at 5000 rpm for 20 min, resuspended in lysis buffer (50 mM Tris–HCl (pH 8.0), 400 mM NaCl, 1 mM TCEP) and lysed by a cell disruptor. The lysate was clarified by ultracentrifugation at 18,000 rpm for 50 min. The supernatant was loaded onto a HisTrap FF column (Cytiva, Marlborough, MA, USA) equilibrated with lysis buffer, washed with lysis buffer and followed by elution using elution buffer (50 mM Tris–HCl pH 8.0, 400 mM NaCl, 500 mM imidazole, 1 mM TCEP) with a linear gradient of imidazole ranging from 0 mM to 500 mM. The fractions of M^pro^-His tag were mixed with GST-PreScission protease-His-tag at a molar ratio of 5:1 to remove the C-terminal His tag. The PreScission-treated M^pro^ was applied to nickel column to remove the GST-PreScission protease-His-tag and protein with uncleaved His-tag. The His-tag cleaved M^pro^ in the flow-through was further purified by size-exclusion chromatography (HiLoad™ 16/60 Superdex 75 (Cytiva, Marlborough, MA, USA)) and stored in 20 mM HEPES pH 7.5, 150 mM NaCl, 1 mM TCEP.

### 2.2. M^Pro^ Inhibition Assay

The M^Pro^ peptide substrate Dabcyl-KTSAVLQSGFRKM-E(Edans)-NH_2_, was purchased from GenScript (Piscataway, NJ, USA). His-tagged SARS1-M^Pro^ was purchased from Sino Biological Inc. (Wayne, PA, USA). All assays were done in a 96-well half area plate (Corning, Corning, NY, USA). Peptide cleavage was measured using 50 nM enzyme. Assays were done in 50 mM HEPES pH 7.5, 150 mM NaCl, 1 mM EDTA, 1 mM DTT. SARS1-M^pro^ and SARS2-M^pro^ were incubated with either buffer or 0–50 µM of ML188 for 20 min. ML188 was purchased from MedChemExpress (Monmouth Junction, NJ, USA, CAT# HY-136259) with 98.35% purity. The reaction was initiated by adding 25 µM peptide substrate, followed by 30 min incubation at 25 °C. Fluorescence was measured at 485 nm with excitation at 340 nm with EnVision 2105 plate reader (Perkin Elmer, Waltham, MA, USA). Experiment was performed in duplicate and the error from global fit with variable hill slope to obtain IC_50_ value is reported.

### 2.3. Protein Crystallization

All crystallization screens tested provided conditions that produced M^pro^ cocrystals. A condition producing large crystals was discovered using the PACT Premier crystal screen (Molecular Dimensions, Maumee, OH, USA), Well E9, containing 20% (*w*/*v*) PEG 3350 and 0.2 M Potassium Sodium Tartrate Tetrahydrate. The SARS2-M^pro^-ML188 cocrystal was grown at room temperature by hanging drop vapor diffusion method in a 24-well VDX hanging-drop tray (Hampton Research, Journey Aliso Viejo, CA, USA) with a protease concentration of 6.0 mg/mL with 6-fold molar excess of ML188 (10% DMSO) and mixed with the precipitant solution at a 1:1 ratio (1 µL:1 µL) and micro-seeded (1:100–1:10,000 dilution) with a cat whisker. Crystals appeared overnight and grew to diffraction quality after 3 days. As data was collected at 100 K, cryogenic conditions consisted of the precipitant solution supplemented with 25% glycerol.

### 2.4. Data Collection and Structure Determination

Diffraction quality crystals were flash frozen under a cryostream when mounted on our in-house Rigaku_Saturn944 X-ray system (Rigaku, The Woodlands, TX, USA). Co-crystal diffraction intensities were indexed, integrated, and scaled using HKL3000 [27]. The structure was solved using molecular replacement with PHASER [28] using an M^pro^ monomer (PDB: 6M03 by Zhang et al. DOI: 10.2210/pdb6M03/pdb). Model building and refinement was performed using Coot [29] and Phenix [30]. During refinement, optimized stereochemical weights were utilized. The ML188 ligand was designed in Maestro and the output SDF file was used in the Phenix program eLBOW [31] to generate the CIF file containing atomic positions and constraints necessary for ligand refinement. Iterative rounds of crystallographic refinement were carried out until convergence was achieved. To limit bias throughout the refinement process, five percent of the data was reserved for the free R-value calculation [32]. MolProbity [33] was applied to evaluate the final structure before deposition in the PDB [34,35]. Structure analysis, superposition and figure generation was done using PyMOL [36]. X-ray data collection and crystallographic refinement statistics are presented in the Supporting Information (Table S1).

### 2.5. Intermolecular vdW Contact Analysis of Crystal Structures

To calculate the intermolecular vdW interaction energies, the crystal structures were prepared using the Schrödinger Protein Preparation Wizard [37]. Hydrogen atoms were added, protonation states were determined, and the structures were minimized. Subsequently, force field parameters were assigned using the OPLS3 force field [38]. Interaction energies between the inhibitor and protease were estimated using a simplified Lennard-Jones potential, as previously described in detail [39]. Briefly, the vdW energy was calculated for pairwise interactions depending on the types of atoms interacting and the distance between them.

## 3. Results

To compare the potency of ML188, we purchased SARS1-M^pro^ and expressed and purified SARS2-M^pro^ for enzyme inhibition assays. Using a FRET-based enzymatic assay, ML188 inhibits SARS1-M^pro^ with an IC_50_ of 4.5 ± 0.5 µM and inhibits SARS2-M^pro^ with an IC_50_ of 2.5 ± 0.3 µM. Therefore, ML188 is approximately twice as potent in SARS2 compared to SARS1 (Figure 1b).

**Figure 2:**
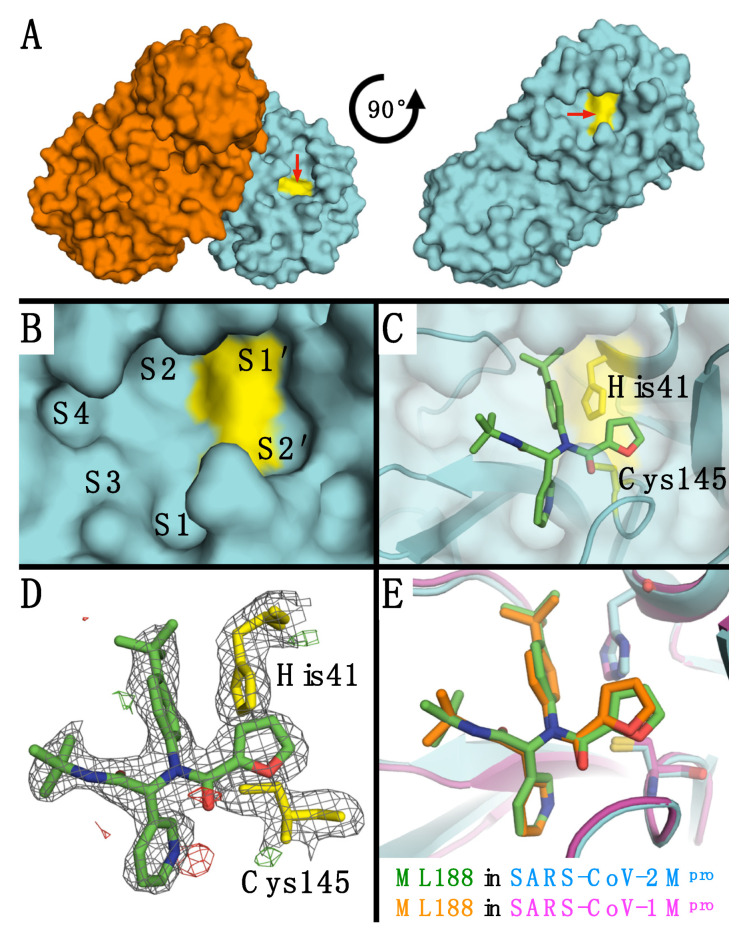
(**A**) SARS2-M^pro^ dimer and single subunit shown in surface representation (rotated on the plane of the page). (**B**) SARS2-M^pro^ active site with subsites labeled and catalytic residues colored yellow. (**C**) ML188 in the SARS2-M^pro^ active site with catalytic residues labeled. (**D**) Electron density around ML188 and catalytic residues. The 2F_o_-F_c_ direct maps are depicted as grey mesh contoured at 1.0 σ while the F_o_-F_c_ difference maps have positive density depicted as green mesh contoured at 3.0 σ and negative density as red mesh contoured at −3.0 σ. (**E**) Alignment of ML188 in SARS2-M^pro^ (teal protein and green ligand; PDB: 7L0D) and SARS1-M^pro^ (magenta protein and orange ligand; PDB: 3V3M).

The SARS-M^pro^ is a functional homodimer (Figure 2A) that binds and cleaves 11 different substrates. The protease has well defined subsites at P1 and P2, which recognize glutamine and large hydrophobic residues, respectively. To elucidate structural differences in ML188 binding, the cocrystal structure of ML188 in complex with SARS2-M^pro^ was determined to 2.39 Å resolution (Table S1; Figure 2). The SARS2-M^pro^-ML188 complex was solved in the same space group (C2) as the SARS1-M^pro^-ML188 complex (PDB: 3V3M). Both structures contain one M^pro^ subunit in the asymmetric unit, but the complexes had different cell dimensions with vastly different crystal packing (Figure S2). The electron density permitted ML188 to be modeled unambiguously in the same orientation as found in the SARS1-M^pro^-ML188 complex (Figure 2D). Overall, ML188 binds similarly in both complexes and makes all the same interactions (Figure 2E), with subtle differences.

**Figure 3:**
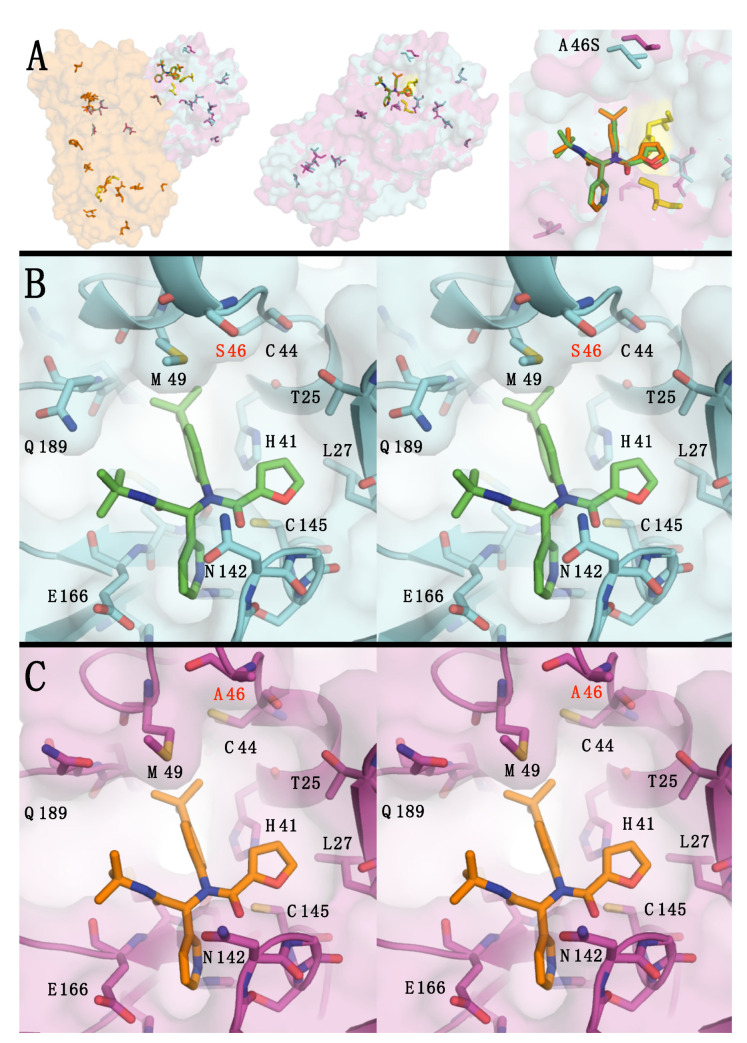
(**A**) The twelve amino acid differences between SARS2 and SARS1-M^pro^ shown as a dimer, single subunit, and active site. SARS2 (in cyan) and SARS1 (in magenta) are shown with transparent surfaces and amino acid differences shown as sticks. The dimer subunits (in orange) and the catalytic residues (in yellow) are included. (**B**) Stereo pair of SARS2-M^pro^-ML188 complex active site. (**C**) Stereo pair of SARS1-M^pro^-ML188 complex active site.

Aligning the SARS1-M^pro^-ML188 and SARS2-M^pro^-ML188 complexes in PyMOL finds the protease alpha carbons have a root-mean-square deviation (RMSD) of 0.53 Å. The largest relevant C-α backbone differences occurred at residues 45–49 in the loop above the S2 subsite (Figure S3). This may relate to the substitution in this loop and the overall 12-residue difference between SARS1 and SARS2 M^pro^ (Figure 3A and Figure S1). The mutation closest to the active site is A46S (Figure 3B,C), which was found to affect the dynamics of that pocket [12]. Changes in protease dynamics can affect the overall binding potency of inhibitors.

The structures were used to calculate protease-inhibitor van der Waals (vdW) contacts that showed minor differences in interactions, SARS1-M^pro^-ML188 with −59.3 kcal/mol versus SARS2-M^pro^-ML188 with −62.0 kcal/mol. This difference of 2.3 kcal/mol is directly due to the conformers modeled into the electron density of three side chains, specifically M49, N142, and Q189 (Figure S4). The vdW calculations also show that ML188 interacts more with the catalytic histidine H41. All other interactions are within the margin of error for structures of this resolution, but the overall trends suggest ML188 packs deeper into the S2 subsite of SARS2-M^pro^, but less deep into the S1 subsite compared to SARS1-M^pro^ (Figure S4).

## 4. Conclusions

In this study, we determine that the non-covalent inhibitor ML188 has enhanced binding potency to SARS2-M^pro^ compared to SARS1-M^pro^. We characterize the complex of SARS2-M^pro^ with ML188 and compare it to the SARS1-M^pro^-ML188 complex. Overall, the complexes are very similar in both proteases, but subtle differences likely contribute to the higher potency of ML188 against SARS2-M^pro^. This indicates ML188 may provide a scaffold for a robust non-covalent pan-coronavirus inhibitor.

## Data Availability

The structural data presented in this study are openly available in the Protein Data Bank, identification number 7L0D. Inhibition data presented in this study are available on request from the corresponding author.

## Supplementary Materials

The following are available online at https://www.mdpi.com/1999-4915/13/2/174/s1, Figure S1: Sequence alignment of SARS2-M^pro^ and SARS1-M^pro^. Table S1: X-ray data collection and crystallographic refinement statistics. Figure S2: Comparison of crystallographic symmetry mates. Figure S3: C-alpha distance differences between SARS2-M^pro^ ML188 complex and SARS1-M^pro^ ML188 complex. Figure S4: Protease-Inhibitor vdW differences.
