# Supplementary material for: Crystal Structure of SARS-CoV-2 Main Protease in Complex with the Non-Covalent Inhibitor ML188
Source: Viruses. 2021 Jan 25;13(2):174. doi: 10.3390/v13020174 (PMC7911568; doi:10.3390/v13020174)
Supplement: Supplementary file 1 [file viruses-13-00174-s001.pdf]

Supplementary Material

Crystal Structure of SARS-CoV-2 Main Protease in Complex with the Non-Covalent Inhibitor ML188

*Gordon J. Lockbaum, Archie C. Reyes, Jeong Min Lee, Ronak Tilvawala, Ellen A. Nalivaika, Akbar Ali, Nese Kurt Yilmaz, Paul R. Thompson, Celia A. Schiffer**

Department of Biochemistry and Molecular Pharmacology, University of Massachusetts Medical School, Worcester, Massachusetts 01605, United States

*Corresponding author. Email: Celia.Schiffer@umassmed.edu

Supplementary Materials

Figure S1: Sequence alignment of SARS2-M^{pro} and SARS1-M^{pro}. Table S1: X-ray data collection and crystallographic refinement statistics. Figure S2: Comparison of crystallographic symmetry mates. Figure S3: C-alpha distance differences between SARS2-M^{pro} ML188 complex and SARS1-M^{pro} ML188 complex. Figure S4: Protease-Inhibitor vdW differences.

```
SARS1     1 SGFRKMAFPSGKVEGCMVQVTCGTTTLNGLWLDDTVYCPRHVICTAEDML   50
            ||||||||||||||||||||||||||||||||||:||||||||||:||||
SARS2     1 SGFRKMAFPSGKVEGCMVQVTCGTTTLNGLWLDDVVYCPRHVICTSEDML   50

SARS1    51 NPNYEDLLIRKSNHSFLVQAGNVQLRVIGHSMQNCLLRLKVDTSNPKTPK  100
            ||||||||||||||:||||||||||||||||||||:|:|||||:||||||
SARS2    51 NPNYEDLLIRKSNHNFLVQAGNVQLRVIGHSMQNCVLKLKVDTANPKTPK  100

SARS1   101 YKFVRIQPGQTFSVLACYNGSPSGVYQCAMRPNHTIKGSFLNGSCGSVGF  150
            |||||||||||||||||||||||||||||||||:||||||||||||||||
SARS2   101 YKFVRIQPGQTFSVLACYNGSPSGVYQCAMRPNFTIKGSFLNGSCGSVGF  150

SARS1   151 NIDYDCVSFCYMHHMELPTGVHAGTDLEGKFYGPFVDRQTAQAAGTDTTI  200
            |||||||||||||||||||||||||||||:||||||||||||||||||||
SARS2   151 NIDYDCVSFCYMHHMELPTGVHAGTDLEGNFYGPFVDRQTAQAAGTDTTI  200

SARS1   201 TLNVLAWLYAAVINGDRWFLNRFTTTLNDFNLVAMKYNYEPLTQDHVDIL  250
            |:||||||||||||||||||||||||||||||||||||||||||||||||
SARS2   201 TVNVLAWLYAAVINGDRWFLNRFTTTLNDFNLVAMKYNYEPLTQDHVDIL  250

SARS1   251 GPLSAQTGIAVLDMCAALKELLQNGMNGRTILGSTILEDEFTPFDVVRQC  300
            ||||||||||||||||:|||||||||||||||||::||||||||||||||
SARS2   251 GPLSAQTGIAVLDMCASLKELLQNGMNGRTILGSALLEDEFTPFDVVRQC  300

SARS1   301 SGVTFQ    306
            ||||||
SARS2   301 SGVTFQ    306
```

Figure S1. Sequence alignment of SARS2-M^{pro} vs SARS1-M^{pro}. Differences include: T35V, A46S, S65N, L86V, R88K, S94A, H134F, K180N, L202V, A267S, T285A, and I286L.

Table S1. X-ray data collection and crystallographic refinement statistics. Values in parentheses are for the highest resolution shell.

	**SARS2-M^{pro}-ML188**
PDB ID	7L0D
**Data Collection**	
Space group	C121
a, b, c (Å)	113.2, 52.7, 46.1
α, β, *γ* (°)	90, 102.8, 90
Resolution (Å)	30.1 - 2.39 (2.47 - 2.39)
Unique Reflections	9338 (886)
Total Reflections	28452 (2586)
Redundancy	3.0 (2.9)
Completeness (%)	87.9 (84.6)
Average I/σ	12.3 (3.4)
R_{merge} [a]	0.069 (0.313)
R_{pim}	0.045 (0.210)
CC 1/2	0.995 (0.862)
**Refinement**	
R_{factor} (%)[c]	20.1
R_{free} (%)[d]	26.1
RMSD[b] in:	
Bond lengths (Å)	0.003
Bond angles (°)	0.550
Ramachandrans:	
Favored (%)	97.36
Allowed (%)	2.31
Outliers (%)	0.33
B-factors:	
Average	35.66
Macromolecules	35.55
Ligands	36.61
Solvent	37.77

[a]$R_{\mathrm{sym}} = \Sigma \mid I - \langle I \rangle \mid / \Sigma I$, where *I* = observed intensity, $\langle I \rangle$ = average intensity over symmetry equivalent

[b]RMSD, root mean square deviation.

[c]$R_{factor} = \Sigma \mid\mid F_o \mid - \mid F_c \mid\mid / \Sigma \mid F_o \mid$.

[d]R_{free} was calculated from 5% of reflections, chosen randomly, which were omitted from the refinement process.

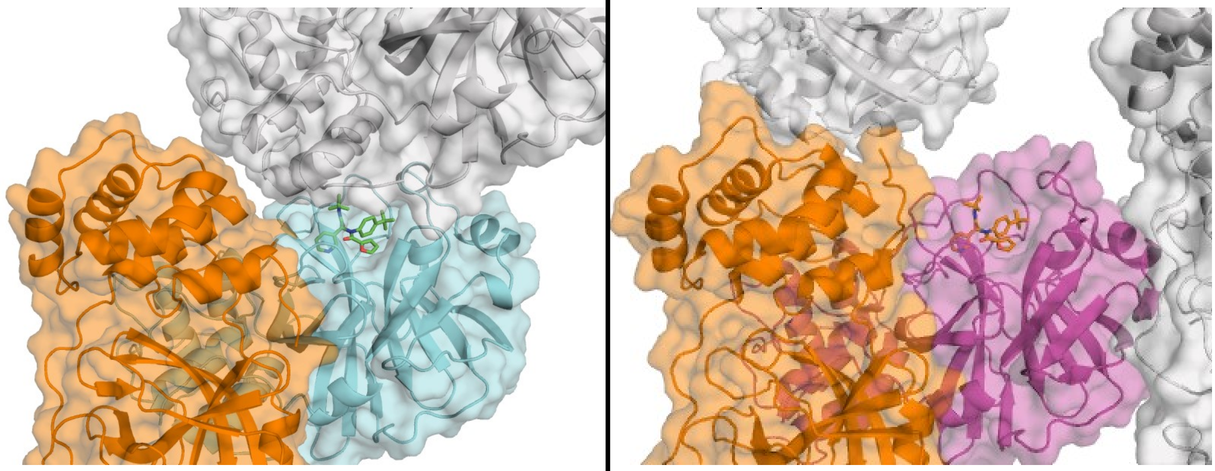

Space Group	Length: a, b, c (Å)	Angles: α, β, γ (°)	Space Group	Length: a, b, c (Å)	Angles: α, β, γ (°)
C121	113.2, 52.7, 46.1	90, 102.8, 90	C121	106.7, 82.7, 53.1	90, 106.0, 90

Figure S2. Comparison of crystallographic symmetry mates. SARS2-M^{pro}-ML188 complex (cyan) and SARS1-M^{pro}-ML188 complex (magenta) with natural dimer (orange) and relevant crystallographic symmetry mates within 4 Å (grey)

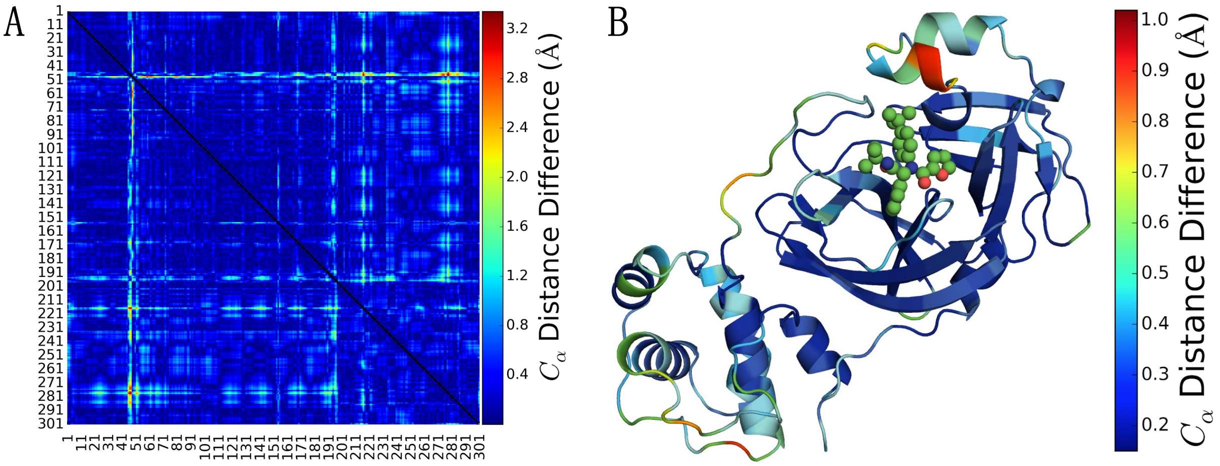

Figure S3. A) C-alpha distance differences between SARS2-M^{pro}-ML188 and SARS1-M^{pro}-ML188. Residues 301-306 were excluded because the differences at those residues were large and overshadowed the analysis. B) Average C-alpha distance differences plotted onto structure, shown as cartoon, with inhibitor ML188 shown as green spheres.

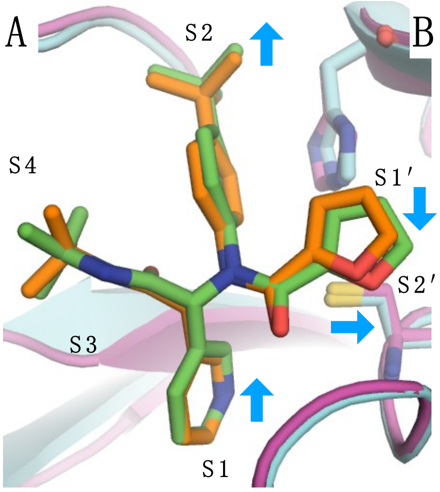

B

Resi	SARS1	SARS2	Diff	Subsite
T25	-0.9	-1.0	-0.1	S2'
T26	-0.8	-0.8	0.0	S2'
L27	-1.5	-1.7	-0.2	S2'
H41	-6.0	-6.5	-0.6	S2
C44	-1.1	-0.8	0.3	S2
M49	-3.8	-4.3	-0.6	S2
Y54	-0.4	-0.5	0.0	S2
F140	-2.0	-1.9	0.1	S1
L141	-2.5	-2.6	-0.1	S1
N142	-5.0	-5.8	-0.8	S1
G143	-2.0	-2.0	-0.1	S1
S144	-1.6	-1.3	0.3	S1
C145	-4.5	-4.2	0.3	S1
H163	-1.9	-1.8	0.1	S1
H164	-2.2	-2.0	0.2	S2
M165	-5.6	-5.4	0.1	S2
E166	-7.7	-7.5	0.1	S3
L167	-0.6	-0.6	0.0	S4
H172	-1.1	-0.9	0.2	S1
D187	-1.6	-1.8	-0.2	S2
R188	-1.8	-2.0	-0.2	S2
Q189	-1.9	-3.1	-1.2	S4

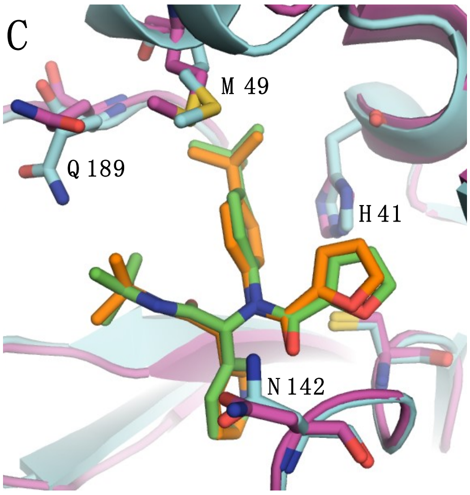

Figure S4. A) Overall shifts in Inhibitor binding between ML188 and SARS2-M^{pro} and SARS1-M^{pro}, emphasized with blue arrows. B) Per-residue protein-inhibitor vdW contacts between ML188 and SARS2-M^{pro} and SARS1-M^{pro}. In the "Diff" column, **Red** indicates ML188 makes more contacts in SARS2 than SARS1, and **Blue** indicates less contacts. C) Residues that showed the largest differences in protein-inhibitor vdW contacts are shown as sticks.
